# Regulation of GAP43/calmodulin complex formation via calcineurin-dependent mechanism in differentiated PC12 cells with altered PMCA isoforms composition

**DOI:** 10.1007/s11010-015-2473-4

**Published:** 2015-06-05

**Authors:** Tomasz Boczek, Bozena Ferenc, Malwina Lisek, Ludmila Zylinska

**Affiliations:** Department of Molecular Neurochemistry, Medical University of Lodz, 6/8 Mazowiecka Str., 92-215 Lodz, Poland

**Keywords:** Calmodulin, GAP43, Calcineurin, Calcium homeostasis, PC12 cells, PMCA

## Abstract

Several lines of evidence suggest the contribution of age-related decline in plasma membrane calcium pump (PMCA) to the onset of neurodegenerative diseases. From four PMCA isoforms, PMCA2, and PMCA3 respond to a rapid removal of Ca^2+^ and are expressed predominantly in excitable cells. We have previously shown that suppression of neuron-specific PMCAs in differentiated PC12 cells accelerated cell differentiation, but increased apoptosis in PMCA2-deficient line. We also demonstrated that altered expression of voltage-dependent calcium channels correlated with their higher contribution to Ca^2+^ influx, which varied between PMCA-reduced lines. Here, we propose a mechanism unique for differentiated PC12 cells by which PMCA2 and PMCA3 regulate pGAP43/GAP43 ratio and the interaction between GAP43 and calmodulin (CaM). Although down-regulation of PMCA2 or PMCA3 altered the content of GAP43/pGAP43, of paramount importance for the regulatory mechanism is a disruption of isoform-specific inhibitory PMCA/calcineurin interaction. In result, higher endogenous calcineurin (CaN) activity leads to hypophosphorylation of GAP43 in PMCA2- or PMCA3-deficient lines and intensification of GAP43/CaM complex formation, thus potentially limiting the availability of free CaM. In overall, our results indicate that both “fast” PMCA isoforms could actively regulate the local CaN function and CaN-downstream processes. In connection with our previous observations, we also suggest a negative feedback of cooperative action of CaM, GAP43, and CaN on P/Q and L-type channels activity. PMCAs- and CaN-dependent mechanism presented here, may signify a protective action against calcium overload in neuronal cells during aging, as well a potential way for decreasing neuronal cells vulnerability to neurodegenerative insults.

## Introduction

Cellular function of plasma membrane calcium pump (PMCA) focuses on Ca^2+^ extrusion and protection from calcium overload, particularly on prevention from cell apoptosis as well on controlling of calcium signaling. The existence of four isoforms encoded by four different genes and tissue-specific composition of PMCAs variants in the membrane domains strongly implicate their unique function in controlling of calcium homeostasis [for ref. 1, 2]. PMCA2 and PMCA3 isoforms are the fastest calcium pumps with prevailing expression in excitable cells, in contrast to ubiquitously expressed PMCA1 and PMCA4, which are thought to perform a housekeeping function [3–5]. However, some data suggest more comprehensive connection of these proteins with internal cellular event, like development, differentiation, secretion or formation of signaling complexes, but not all PMCA isoforms appear to be involved to the same extent [6, 7]. Although PMCA is a solely ion pump directly activated by Ca^2+^/CaM complex, the affinity and rate of activation varies considerably between particular isoforms [1].

Among many players, calmodulin (CaM), calcineurin (CaN), and neuromodulin (GAP43) are strongly linked with calcium. Moreover, the availability of CaM is controlled by its interaction with neuromodulin [8–10]. GAP43 is synthesized in the neuronal cell body and becomes highly enriched in the tips of extending neurites. The local mobility of GAP43 in a temporally and spatially defined manner depends on posttranslational modifications of protein, and phosphorylation at Ser-41 by PKC appears to be functionally crucial, since it markedly reduces GAP43 affinity for CaM [11–13]. Dephosphorylation of GAP43 by calcineurin increases formation of CaM/GAP43 complex and, as a consequence, it potentially declines the amount of Ca^2+^/CaM [14]. It should be noted that both, PMCA and calcineurin are the effectors for Ca^2+^/CaM action.

PC12 cells derive from a transplantable rat pheochromocytoma and are proliferating cells, which synthesize, release, take up, and store catecholamines [15]. A notable feature of PC12 cells is that upon the neurotrophin exposure they cease proliferation and differentiate into sympathetic-like neurons, become electrically excitable, express neuronal markers and extend neurites [16]. Therefore, it is one of the most frequently used models for studying the neuronal processes occurring during differentiation. Non-differentiated PC12 cells expressed primarily PMCA 1b, 2b, 3a, 3b, 3c, and 4b, but after differentiation mRNAs of the variants 1c, 2a, 2c, and 4a were also identified [17, 18].

In our previous studies, we established stable transfectans of non-differentiated PC12 cells with down-regulated expression of PMCA2 (_2 line) or PMCA3 (_3 line) to elucidate the PMCAs role in cell development [19, 20]. The main observation was higher intracellular Ca^2+^ concentration in PMCA-reduced lines, lowered total calcium pump activity, but also decreased proliferation rate and triggering of early neuritogenesis. Moreover, we found several important changes in the expression of number of genes, including GAP43, CaM, and CaN, as well corresponding proteins. Functional studies have shown that reduction in PMCA2 or PMCA3 led to Ca^2+^-dependent activation of calcineurin/NFAT signaling and, in consequence, to the inhibition of dopamine secretion and deregulation of the secretory apparatus [21–23]. From these results, we concluded that PMCA2 and PMCA3 are not only simple ion transporting proteins, but are a part of more complex system regulating vital cell functions.

Therefore, we next developed differentiated PC12 cell lines with stable down-regulated expression of PMCA2 (_2 line) or PMCA3 (_3 line) to analyze the consequences of altered calcium homeostasis in neuron-like cells [24]. We have shown that experimental reduction in the PMCA2 or PMCA3 content by almost 50 % elevated cytosolic Ca^2+^ concentration from ~92 nM in control cells to ~0.149 and ~130 nM in _2 and _3 lines, respectively. Furthermore, the accelerated differentiation and formation of longer neurites in PMCA-reduced lines were observed, but increased population of apoptotic cells was noted in _2 line. Functional studies revealed altered expression of certain types of voltage-gated calcium channels (VGCCs) in PMCA-reduced cells, which correlated with their higher contribution to Ca^2+^ influx [25]. We have also evidenced a novel role of PMCA isoforms in regulation of bioenergetic pathways and mitochondrial activity [26].

Taking into consideration that PMCA amount and activity decrease with aging [27] but intracellular Ca^2+^ is an important regulator of cell survival and cell death, these results gave us the background for further analyzes of relationship between CaM, GAP43, and CaN in the neuronal-type of cells with disturbed calcium homeostasis. In contrast to non-differentiated cells, we found more intense CaM/GAP43 complex formation in PMCA2- or PMCA3-deficient lines. This enhanced interaction was fully CaN-dependent, and was also associated with altered binding of CaN with PMCA isoforms 2 and 4.

## Materials and methods

### Reagents

If not otherwise state, all the reagents were purchased in Sigma-Aldrich (Germany). The PC12 rat pheochromocytoma cell line was obtained from ATCC (USA). RPMI 1640 medium was from PAA (Austria). Calf and horse sera were from BioChrom (UK). Alexa Fluor 488, Alexa Fluor 594 and M-MLV Reverse Transcriptase were from Life Technologies (USA). Protein Assay Kit was from Bio-Rad (USA). Total RNA isolation kit was from Epicentre Biotech. (USA). Maxima SYBR Green Master Mix was from Fermentas (Canada). Primary antibodies against calcineurin, GAP43, phosphoGAP43, GAPDH were from Santa Cruz Biotech. (USA). Primary antibodies against calmodulin, PMCA1, PMCA2, PMCA3, PMCA4 were purchased in Thermo Scientific (USA). Calcineurin cellular activity assay kit was from Enzo Life Sciences (USA). Primers were synthesized in the Institute of Biochemistry and Biophysics (Poland).

### Cell culture

C-terminal sequences of PMCA2 or PMCA3 were cloned in the antisense orientation into pcDNA3.1(+) vector as described previously [19]. The obtained anti-PMCA2 or anti-PMCA3 constructs were subsequently transfected into PC12 cells using TurboFect^TM^ Transfection Reagent according to the manufacturer’s instructions. The transection procedure was repeated 24 h later. Stable transfectants were selected using G418 at concentration of 600 μg/ml for 2 weeks and then, the concentration of antibiotic was reduced to 200 μg/ml. This concentration of selective agent was continuously present in the growth medium to ensure constant presence of anti-PMCA2 or anti-PMCA3 vectors in the cells, and thus an expression of antisense mRNA. Nevertheless, the expression of *Atp2b2* and *Atp2b3* encoding PMCA2 and PMCA3, respectively, was controlled by real-time PCR and the level of protein product was assessed by Western blot every 3 passages. Mock-transfected PC12 cells carrying G418 resistance were used as a control. Cells were routinely grown on collagen (type I from rat tail)-coated flasks in RPMI 1640 medium containing 10 % horse serum, 5 % fetal bovine serum, 25 mM HEPES, pH 7.4, 2 mM l-glutamine, 1 mM sodium pyruvate, 25 U/ml penicillin, 25 μg/ml streptomycin at 37 °C in 5 % CO_2_ in a humidified incubator. For most of the experiments, cells were plated at a density of 1–2 × 10^5^ cells/ml.

### Cell differentiation

Obtained stably transfected lines were differentiated with 1 mM dibutyryl-cAMP (db-cAMP) added 48 h following seeding and the cells were maintained in the presence of differentiating agent for another 48 h. No more than 17 passages were used. In some experiments, the inhibitor of calcineurin—cyclosporin A (CsA) at a final concentration of 10 μM—was added prior to differentiation and was present throughout the differentiation period.

### Preparation of total cell lysate

Cells were lysed in RIPA buffer supplemented with 1 mM PMSF, 2 mM Na_3_VO_4,_ and protein inhibitor cocktail (10 μg/ml) for 30 min on ice. Nuclei and cellular debris were separated by centrifugation at 10,000×*g* for 20 min. The supernatant was collected and boiled in the Laemmli buffer for 5 min to obtain total cell lysate. Protein concentration was measured using Bio-Rad protein Assay.

### Western blot analysis

For Western blot, 40–60 μg or 80 μg (for phosphoprotein detection) of protein lysate were resolved on a 8 % SDS-PAGE and transferred onto nitrocellulose membrane using a semi-dry method. Membranes were first blocked with 3 % bovine serum albumin (BSA) in TBS-T (10 mM Tris–HCl, pH 7.4, 150 mM NaCl, 0.05 % Tween-20) for 1 h at room temperature and then overnight incubated with primary antibodies at 4 °C. The following primary antibodies were used: polyclonal anti-PMCA1, anti-PMCA2, anti-PMCA3 (diluted 1:1000), monoclonal anti-PMCA4 (1:1000), polyclonal anti-GAP43 (1:1000), polyclonal anti-phosphoGAP43 (1:750), monoclonal anti-calmodulin (1:1500), and polyclonal anti-calcineurin A (1:1000). Polyclonal anti-GAPDH (1:1000) antibodies were used to standardize each line and as an integral loading control. Following 3 × 15 min washes with TBS-T, membranes were incubated for 2 h at room temperature with species-specific secondary antibodies (1:5000) conjugated with alkaline phosphatase. Bands were visualized with BCIP/NBT used according to the manufacturer’s protocol. Blots were densitometrically quantified using GelDocEQ with Quantity One 1-D Analysis Software version 4.4.1 (Bio-Rad).

### Quantitative real-time PCR

Total cellular RNA was extracted using Total RNA Isolation Kit following the protocol provided by the manufacturer. Single-stranded cDNA was synthesized using M-MLV Reverse Transcriptase from 1 μg of total RNA with oligo (dT) starters in the optimal conditions recommended by the supplier. The cDNA templates were next used for quantification of gene expression level in real-time PCR reaction using Maxima SYBR Green Master Mix and the gene-specific primers listed in Table 1. The reaction conditions were as follows: 10 min at 95 °C and 40 cycles at 95 °C for 15 s, 60 °C for 30 s and 72 °C for 30 s. The amplification and quantification was performed on an AbiPrism™ 7000 sequence detection system (Applied Biosciences). The C_t_ value was determined automatically by the accompanying software. The fold change was calculated from ΔΔ*C*_t_ according to the comparative $2^{{ - \Delta \Delta C_{\text{t}} }}$ method [28] using *Gapdh* as a reference housekeeping control. The melting curve was run for each set of primers. The primers were either designed with Primer Designing Tool (available at www.ncbi.nlm.nih.gov) or their sequence is available elsewhere.

**Table 1:** Primers used in real-time reactions

Gene	Protein product	Primer sequence	NCBI number
*Atp2b2*	PMCA2	F: 5′-ACCGTGGTGCAGGCCTATGT-3′; R: 5′-GGCAATGGCGTTGACCAGCA-3′	NM_012508.5
*Atp2b3*	PMCA3	F: 5′-AGGCCTGGCAGACAACACCA-3′ R: 5′-TCCCACACCAGCTGCAGGAA-3′	NM_133288.1
*Gap43*	GAP43	F: 5′-GGAATAAGGATCCGAGGAGGAAAGGAG-3′ R: 5′- CTTAAAGTTCAGGCATGTTCTTGGT-3′	NM_017195
*Ppp3ca*	CaN A	F: 5′-AATTTGTTGTCCATACTCCGA-3′ R: 5′- TGTTCATCACGTTGTTCTCG-3′	NM_017041.1
*Gapdh*	GAPDH	F: 5′-GGTTACCAGGGCTGCCTTCT-3′ R: 5′-CTTCCCATTCTCAGCCTTGACT-3′	NG_028301.1

Sequences of primers were taken from [21] and [24]

### Confocal microscopy

Approximately 5 × 10^4^ cells were seeded on poly-l-lysine (50 μg/l) coated glass LabTek II CC2 chamber slides and differentiated with db-cAMP for 48 h. Next, cells were rinsed 3 × 15 min with PBS and fixed with 3.8 % paraformaldehyde for 30 min at room temperature. Following three washes with PBS, cells were permeabilized with 0.05 % Triton X-100 for 15 min at 4 °C and blocked with 6 % BSA for 2 h at room temperature. The coverslips were then overnight incubated with primary antibodies at 4 °C. The following antibodies were used: monoclonal anti-PMCA 5F10 clone (1:150) recognizing all PMCA isoforms, polyclonal anti-GAP43 (1:100), polyclonal anti-phosphoGAP43 (1:100), and monoclonal anti-calmodulin (1:200). Cells were subsequently probed for 3 h at room temperature with secondary antibodies conjugated with AlexaFluor 488 (for Gap43 and phosphoGAP43) or AlexaFluor 594 (for PMCA and calmodulin) both diluted at 1:1000. At final, nuclei were stained with DAPI and the coverslips were mounted using Vectashield mounting medium. The images were taken on TCS SP5 confocal laser-scanning microscope equipped with 63× objective (Leica). The degree of colocalization was assessed with Leica LAS AF Lite software (Leica).

### Co-immunoprecipitation assay

Protein A/G PLUS-Agarose beads were first washed 3 times with PBS, blocked with 6 % BSA for 1 h at 4 °C and then spun down at 14000×*g* for 5 min at 4 °C. Cells were lysed in RIPA buffer as described in the “Preparation of total cell lysate” section. The lysate containing 500 mg of protein was made up to 500 μl of volume with 10 mM Tris–HCl, pH 7.4 and was subsequently pre-cleaned on agarose beads for 2 h at 4 °C and centrifuged at 14000x*g* for 5 min. The supernatant was incubated with polyclonal anti-GAP43 or polyclonal anti-calcineurin (5 μg of antibodies/500 mg of protein) antibodies for 2 h at 4 °C. Next, 20 μl of protein A/G-agarose beads were added and the mixture was overnight incubated at 4 °C. Then, the complexes of agarose beads/antibodies/examined protein were centrifuged at 14,000×*g* for 5 min, the pellets were suspended in the Laemmli buffer, boiled for 5 min, electrophoresed and transferred onto nitrocellulose as described in the “Western blot analysis” section. The membrane containing immunoprecipitated calcineurin was probed using polyclonal anti-PMCA1 (1:1500), polyclonal anti-PMCA2 (1:1000), polyclonal anti-PMCA3 (1:1000), or monoclonal anti-PMCA4 (1:1500) antibodies. When GAP43 was immunoprecipitated, membranes were probed with monoclonal anti-calmodulin antibodies (1:1000). Bands were visualized as described in the "Western blot analysis" section.

### Calcineurin activity

Calcineurin activity was determined using commercial Calcineurin cellular activity assay kit according to the manufacturer’s instructions. In brief, ~5 × 10^7^ cells were lysed at 4 °C for 40 min in a lysis buffer containing 50 mM Tris–HCl, pH 7.4, 100 mM EDTA, 100 mM EGTA, 0.2 % NP-40, 1 mM DTT and protein inhibitor cocktail (10 μg/ml). The first negative sample was a reaction buffer with 1 mM EGTA (inhibiting only Ca^2+^-dependent PP2B) whereas in the second negative control, a reaction buffer was supplemented with 5 μM okadaic acid (inhibitor of PP1 and PP2A). A positive control was human recombinant calcineurin (40 U). All analyzed samples contained 10 μg of isolated proteins and a buffer for the reaction with calmodulin. Next, the phosphopeptide R II was added to all samples which were further incubated for 10 min at 30 °C. The reaction was stopped with Biomol Green™ reagent. The absorbance of the sample was read after 30 min at 620 nm. The concentration of inorganic phosphate liberated during the reaction was calculated based on calibration curve taking into account the background value and values for negative controls. Calcineurin activity was expressed as nmol* P*_*i*_/μg protein/min.

### Statistical analysis

The data are presented as mean ± SEM of n experiments (*n* ≥ 3). Statistical analyses were performed using STATISTICA 8.0 (StatSoft). For multiple comparisons one-way ANOVA with Bonferroni’s correction was used. Two-way ANOVA was applied to compare the effects of 10 μM CsA between all three examined lines. *P* value < 0.05 was considered as statistically significant.

## Results

### The model PC12 lines

**Fig. 1:**
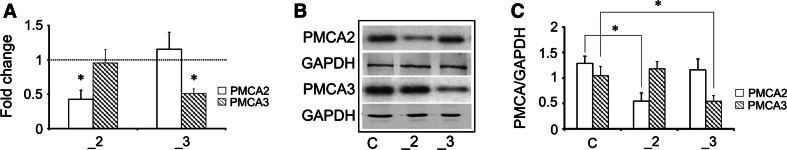
The efficiency of PMCA isoforms knock-down in differentiated PC12 cells. **a** The expression level of PMCA2 and PMCA3 was assessed using real-time PCR (*n* = 5). The results are presented as a relative fold change obtained following normalization to Gapdh expression and calculated using comparative $2^{{ - \Delta \Delta C_{\text{t}} }}$ method. The expression of a target gene in control line was taken as 1 and is presented as a *dotted line*. **b** PMCAs protein was determined with immunoblotting, *n* = 3, and **c** the intensity of bands was quantified densitometrically. The results were normalized to endogenous GAPDH level and are presented as relative units. *P < 0.05, PMCA-deficient lines versus control cells. *C* control line, *_2* PMCA2-deficient line, *_3* PMCA3-deficient line

The model used in our experiments was established several years ago, nonetheless, before we initiated new series of assay, the efficiency of stable transfection with anti-PMCA2 or anti-PMCA3 constructs in differentiated PC12 cell line was validated. Real-time PCR results showed down-regulation of both PMCA isoforms (Fig. 1a), and this was confirmed by Western blot at a protein level (Fig. 1b). Quantification by densitometric analysis showed reduction of PMCA2 and PMCA3 proteins by nearly 50 % in both modified PC12 cell lines in comparison to mock-transfected cells (Fig. 1c).

### Expression of GAP43

**Fig. 2:**
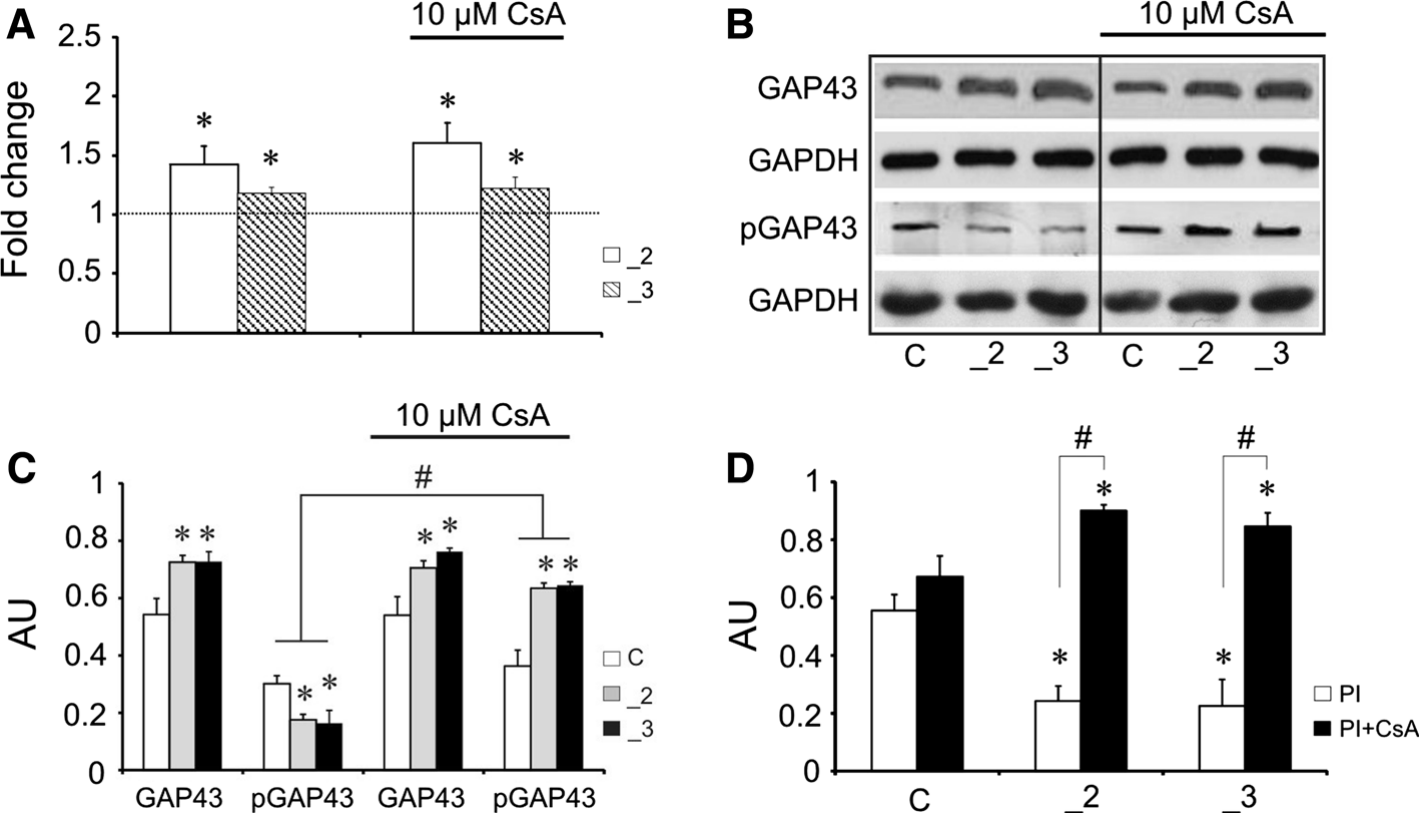
The analysis of GAP43 in PMCAs-deficient lines. **a** GAP43 expression assessed using real-time PCR, *n* = 4. The results are presented as relative units obtained after normalization to Gapdh expression and calculated using comparative $2^{{ - \Delta \Delta C_{\text{t}} }}\;$ method. The expression of a target gene in control line was taken as 1 and is presented as a *dotted line*. **b** Immunocharacteristic of GAP43 and its phosphorylation level, *n* = 4. Representative blots are shown. **c** Densitometrical quantification of bands intensity. The results are presented as arbitrary units (AU) obtained after normalization to endogenous GAPDH level. **d** The index of GAP43 phosphorylation (PI) calculated as pGAP43/GAP43 based on quantified Western blot data presented in **c**. *P < 0.05, PMCA-deficient lines versus control cells. ^#^ *P* < 0.05, CsA-treated versus non-treated lines. *C* control line, *_2* PMCA2-deficient line, *_3* PMCA3-deficient line

Differentiation process is correlated with higher expression of GAP43, a protein being the cellular marker of neuronal development [8]. As shown in Fig. 2 the presence of GAP43 was analyzed using different methods. Real-time PCR demonstrated the increase in mRNA amount in both PMCA-reduced lines when compared to the control cells (Fig. 2a). The presence of cyclosporin A did not change the expression level in all examined PC12 lines. The Western blotting revealed higher amount of immunoreactivity in the PMCA-reduced cell lysates probed with anti-GAP43, an antibody recognizing total GAP43 protein, and the same results were obtained in the presence of CsA (Fig. 2b, c). Interestingly, in PMCA-reduced lines lowered pGAP43 level was detected in the absence of CsA, but it significantly increased after treatment with CsA (Fig. 2b, c). Although total GAP43 level in all examined cell lines was unchanged by CsA presence, the phosphorylation index altered markedly (Fig. 2d). It could indicate that in lines _2 and _3 without CsA treatment more GAP43 protein exists in dephosphorylated form, which may potentially bind calmodulin.

### Colocalization of GAP43 with PMCA and calmodulin

**Fig. 3:**
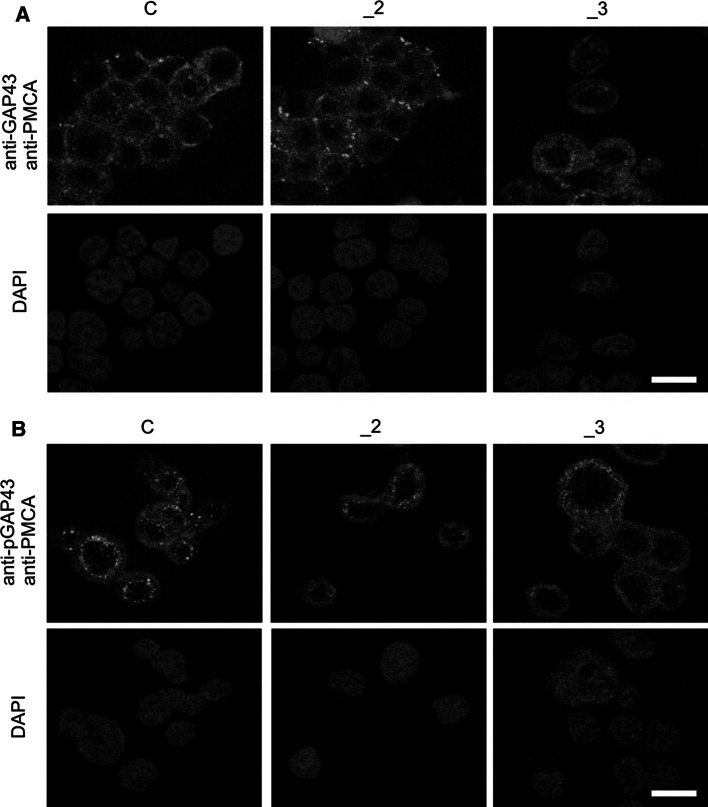
GAP43 and PMCA in PMCAs-deficient lines. Representative confocal fluorescent images illustrating **a** GAP43 and PMCA or **b** pGAP43 and PMCA co-staining in differentiated PC12 lines. GAP43 or pGAP43 (*green*) was labeled using protein-specific primary antibodies and anti-rabbit secondary antibodies conjugated with AlexaFluor 488. PMCA (*red*) was labeled with primary 5F10 antibodies recognizing all isoforms and anti-mouse secondary antibodies conjugated with AlexaFluor 594. Nuclei (*blue*) were stained with DAPI. *Scale bar* 20 μm. *C* control line, *_2* PMCA2-deficient line, *_3* PMCA3-deficient line. (Color figure online)

Since GAP43 in the plasma membrane preferentially exists in specific areas—rafts, and PMCA—was also shown to be localized with these domains [8, 29], we next analyzed the co-localization of both proteins. Results from immunofluorescence combined with confocal microscopy revealed that in all examined cell lines neither GAP43 (Fig. 3a) nor pGAP43 (Fig. 3b) shared the cellular localization with PMCA (~5 and ~3 % for GAP43 and pGAP43, respectively). It suggests lack of direct interaction between these proteins.

**Fig. 4:**
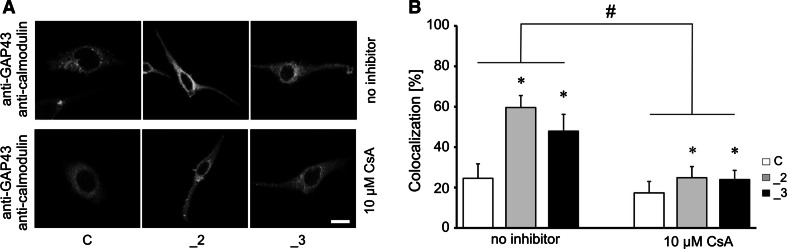
Colocalization of GAP43 and calmodulin. **a** GAP43 was stained with anti-GAP43 primary antibodies and with secondary IgG AlexaFluor 488 (*green*); calmodulin was stained with anti-calmodulin primary antibodies and with secondary IgG AlexaFluor 594 (*red*). Scale bar 20 μm. Representative images are presented. **b** Colocalization was analyzed by Leica LAS AF Lite accompanying software, *n* = 8. *P < 0.05, PMCA-deficient lines versus control cells. ^#^ *P* < 0.05, CsA-treated versus non-treated lines. *C* control line, *_2* PMCA2-deficient line, *_3* PMCA3-deficient line. (Color figure online)

**Fig. 5:**
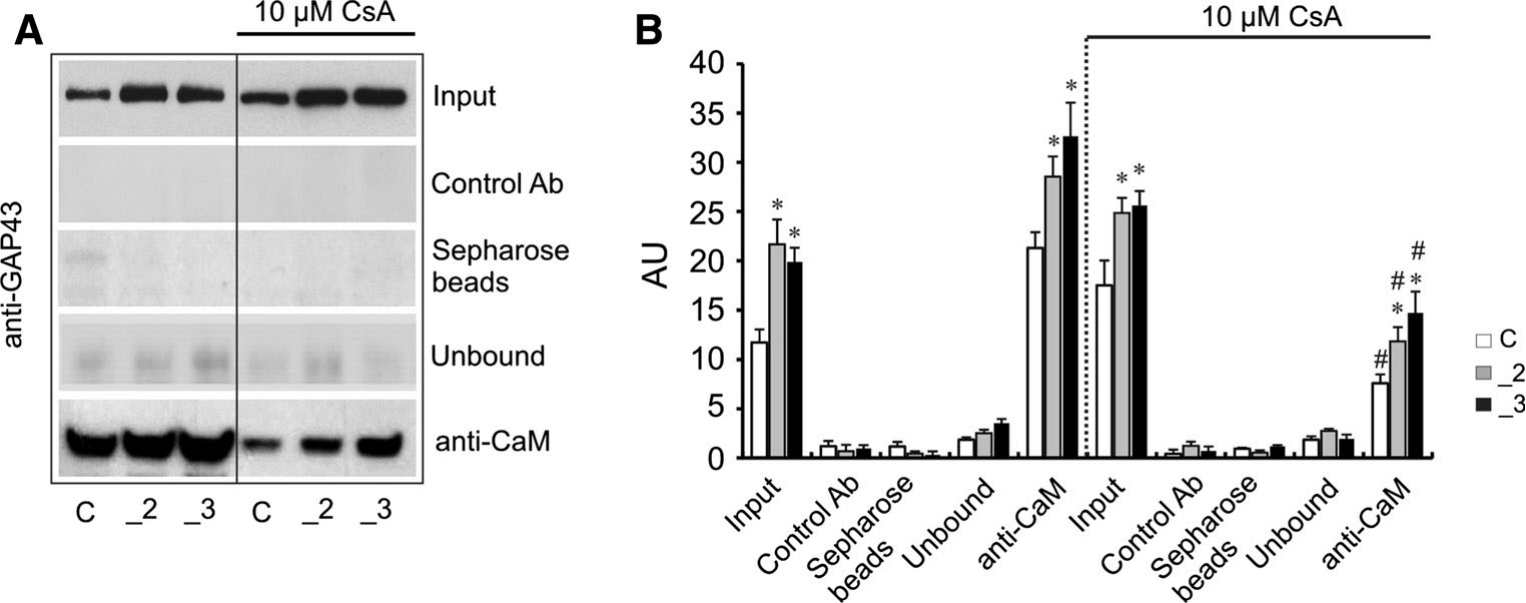
Co-immunoprecipitation of GAP43 and calmodulin (CaM). **a** Lysates from PC12 lines were used to measure GAP43 protein level (input) in non-inhibitory condition and in the presence of cyclosporine A (CsA). Endogenous GAP43 and CaM were co-immunoprecipitated using the anti-GAP43 (7B10) and anti-calmodulin antibodies. Negative controls were done by immunoprecipitating cells with Sepharose-linked secondary antibodies (Control Ab) or only Sepharose beads. Immunoblot analysis also included GAP43 in the supernatant fraction (unbound). Representative blot is shown. **b** Quantitative densitometric analysis of bands intensity. The results are presented as arbitrary units (AU) defined as the optical density per mg of protein (OD/mg protein), *n* = 3. *P < 0.05, PMCA-deficient lines versus control cells. ^#^ *P* < 0.05, CsA-treated versus non-treated lines. *C* control line, *_2* PMCA2-deficient line, *_3* PMCA3-deficient line

One of the physiological functions of GAP43 is controlling of CaM availability. In response to elevation of intracellular calcium or GAP43 phosphorylation, free CaM is released locally [30]. Moreover, PMCA is a sole ion pump directly activated by calmodulin [1]. Thus, we additionally analyzed if GAP43 and calmodulin reside at the same physical location in the cells. In comparison with control cells, PMCA-reduced lines showed 2 times higher colocalization between two fluorescently labeled proteins (Fig. 4a). The similar analysis in the presence of CsA revealed significant decrease in colocalization degree, although in PMCA-reduced lines it was still higher than that of control cells (Fig. 4b). Ability to form complex between GAP43 and CaM was also investigated by co-immunoprecipitation assay. A higher presence of complexes was detected in _2 and _3 lines (Fig. 5a), as well inhibitory effect of CsA on formation of GAP43/CaM complex was observed, confirming contribution of calcineurin to this regulation (Fig. 5b).

### Expression of calcineurin

**Fig. 6:**
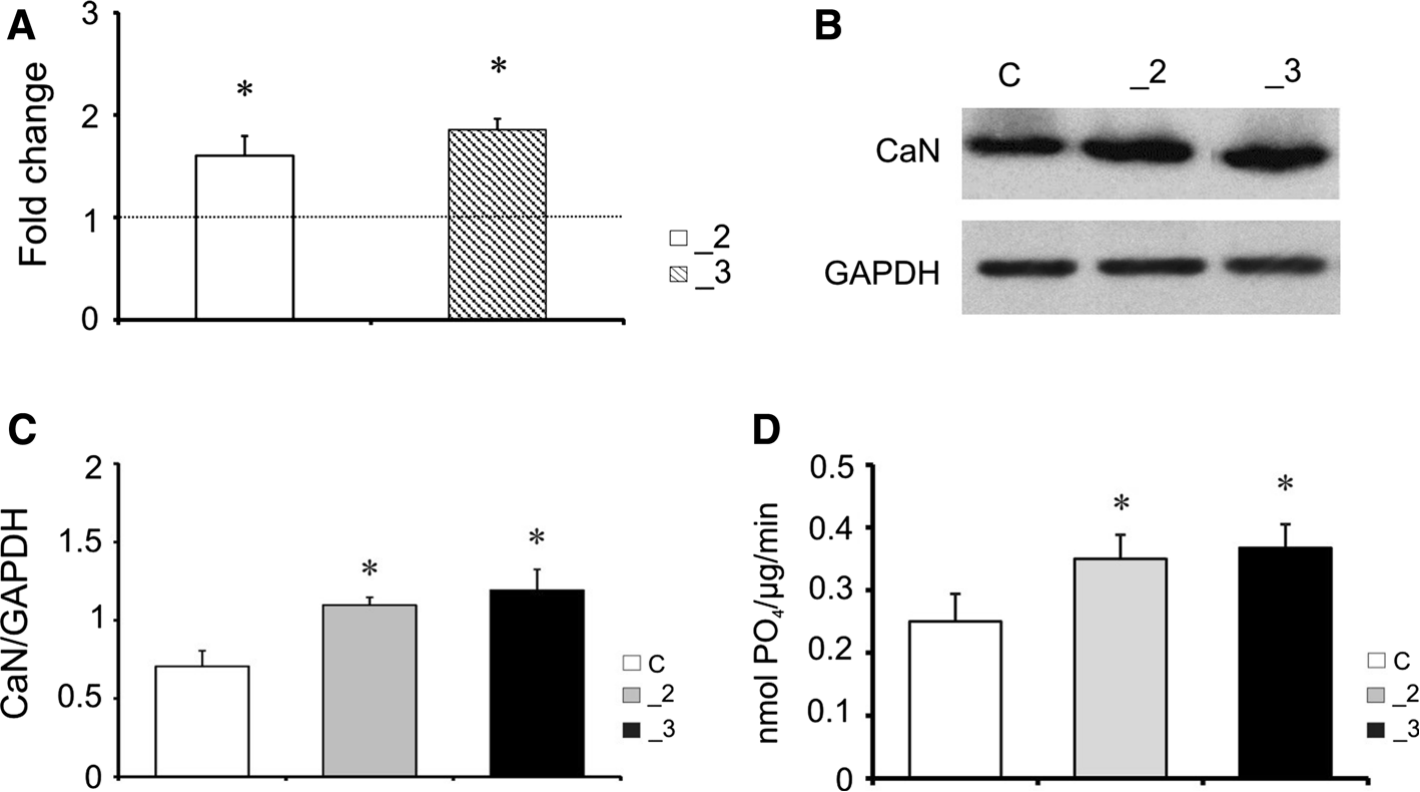
Up-regulation of calcineurin (CaN) in PMCAs-deficient lines. **a** The expression of calcineurin was assessed using real-time PCR (*n* = 4). The results are presented as a relative fold change obtained following normalization to Gapdh expression and calculated using comparative $2^{{ - \Delta \Delta C_{\text{t}} }}\;$method. The expression of a target gene in control line was taken as 1 and is presented as a dotted line. **b** Calcineurin protein level was determined by immunoblotting and **c** the intensity of bands was quantified densitometrically, *n* = 3. The results are presented as relative units obtained after normalization to endogenous GAPDH level. Representative blots are shown. **d** Calcineurin activity determined with Calcineurin Phosphatase Assay Kit as described in the “Materials and methods” section. *P < 0.05, PMCA-deficient lines versus control cells. *C* control line, *_2* PMCA2-deficient line, *_3* PMCA3-deficient line

Calcineurin appears to be a main enzyme responsible for phosphorylation level of GAP43. Therefore, in the next experiments we assayed the amount and activity of this phosphatase. As shown in Fig. 6a, both PMCA-deficient lines exhibited higher expression of CaN mRNA in relation to the control cells. Moreover, the augmented level of protein determined by Western blotting (Fig. 6b, c) correlated with enhanced phosphatase activity (Fig. 6d).

### Co-immunoprecipitation of CaN with PMCA isoforms

**Fig. 7:**
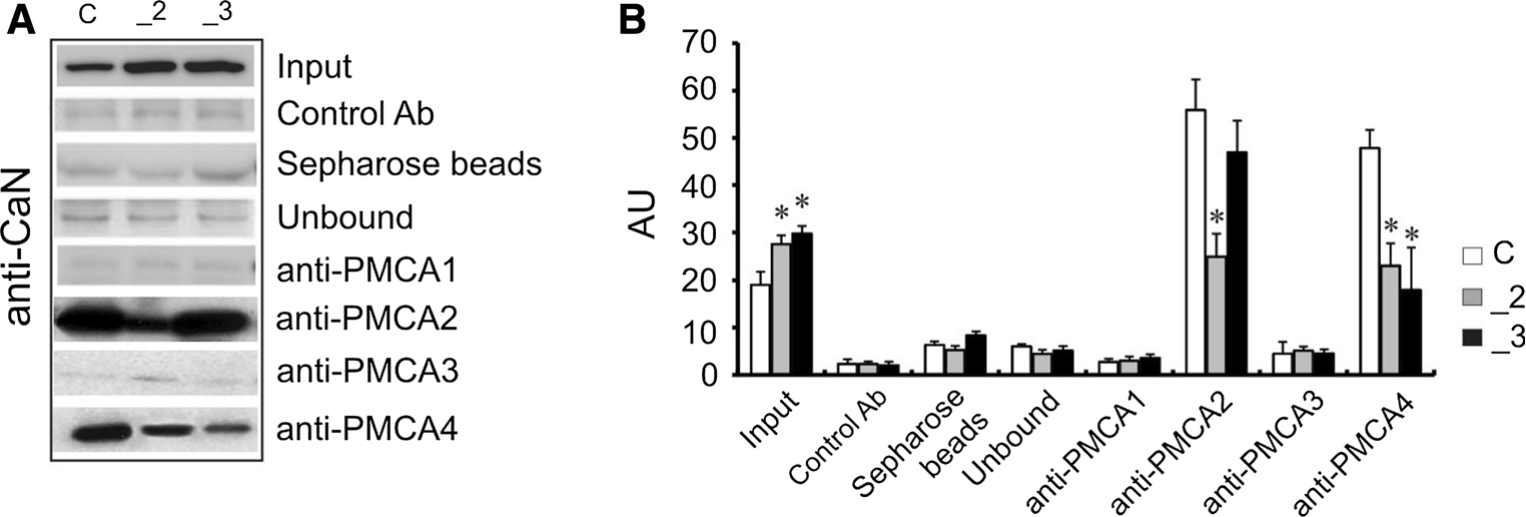
Interaction of calcineurin and PMCA isoforms in PMCA-deficient lines. **a** Cell lysates were incubated with anti-CaN antibodies conjugated to Sepharose beads. Immunocomplexes were resolved by SDS-PAGE and then anti-PMCA1, anti-PMCA2, anti-PMCA3, anti-PMCA4 antibodies were used to detect particular PMCA isoforms with Western blot. Representative blots are shown. Negative controls included Sepharose-linked secondary antibodies (Control Ab) or only Sepharose beads. CaN was also immunoprecipitated in beads-unbound fraction (unbound). **b** Quantitative densitometric analysis of bands intensity. The results are presented as arbitrary units (AU) defined as the optical density per mg of protein (OD/mg protein), *n* = 3. *P < 0.05, PMCA-deficient lines versus control cells. *C* control line, *_2* PMCA2-deficient line, *_3* PMCA3-deficient line

Several studies indicated that CaN can interact with PMCA2 and PMCA4 isoforms, resulting in inhibition of the calcineurin/NFAT signaling pathway [31]. Thus, we subsequently examined the formation of CaN/PMCA complexes using antibodies specific for PMCA isoforms. As was expected, neither PMCA1 nor PMCA3 can bind CaN (Fig. 7). A similar level of CaN/PMCA2 complex was visible in control and _3 cells, and because of PMCA2 reduction, the intensity of immunosignal was decreased by ~55 % in _2 line. Interestingly, PMCA4 scarcely interacted with CaN in both PMCA-reduced lines. When compared with mock-transfected cells, the immunosignal was lowered by ~51 and by ~62 % in _2 and _3 lines, respectively.

## Discussion

Our results provide novel information about the relationship between altered calcium homeostasis and selected Ca^2+^-dependent cellular components—CaM, GAP43, and CaN—which are also responsible for self-regulation of Ca^2+^-sensitive events in the cells. We have previously shown that in differentiated PC12 cells reduction of one of the fast-reacting PMCA isoforms, PMCA2 or PMCA3, resulted in the sustain increase in cytosolic Ca^2+^, more significantly in line _2 [24]. In line with the previous studies on non-differentiated PC12 lines, the following changes were reproduced in both PMCA-reduced differentiated lines: (i) higher content of total GAP43 protein, (ii) lowered pGAP43 level, and (iii) increased amount and activity of calcineurin. This indicates that up-regulation of GAP43 and CaN is not attributed to differentiation process, but is a direct result of PMCA2 or PMCA3 down-regulation. However, in contrast to undifferentiated cells, in this study, we revealed considerably enhanced interaction between GAP43 and CaM, which was dependent on CaN over-activation in PMCAs-deficient lines. This phenomenon, in turn, seems to involve disrupted inhibitory PMCA4-CaN and PMCA2-CaN interaction. The plausible effect of the release of such inhibitory brake is increased cellular CaN phosphatase activity, consequently affecting GAP43/pGAP43 ratio. In these conditions, more CaM can be stored bound to GAP43 what we observed in both PMCAs-reduced lines. Because no such findings were detected in non-differentiated PC12 cells, the results presented here point out a unique mechanism existing in differentiated cells, which may regulate local CaM availability and thus the activity of CaM-dependent downstream targets.

It is well-documented that Ca^2+^/CaM is a very strong modulator of many functional proteins, including the enzymes—plasma membrane calcium pump and calcineurin [32]. One of the main substrates for calcineurin is the transcriptional nuclear factor of activated T-cells (NFAT) and Ca^2+^/CaM-stimulated calcineurin dephosphorylates NFAT enabling its transfer to the nucleus [10]. So far, it has been demonstrated that Ca^2+^/calcineurin-dependent activation of NFAT is important in many processes including neuronal cell differentiation, axonal growth and neuronal development [33, 34].

Interestingly, in several studies CaN has been shown to interact directly with PMCA2 and PMCA4 isoforms, what resulted in the inhibition of CaN-regulated processes [35, 36]. The particular role of PMCA2-dependent inhibition of calcineurin/NFAT pathway was postulated in breast cancer cells, where this association protected cells from apoptosis [31, 37]. In HEK cells, PMCA4b has been shown to inhibit calcineurin/NFAT pathway in a similar manner [38]. On the other hand, the expression of PMCA4b in neurons was shown to be controlled by CaN [39].

In non-differentiated PC12 cells with down-regulated PMCA isoforms, we have observed enhanced interaction of PMCA4 with calcineurin, which resulted in the reduction of dopamine secretion [21]. In our model of differentiated cells, we detected strong interaction between PMCA2 and CaN. The comparative level of PMCA2/CaN co-localization was observed in control and _3 line, but in line _2, because of PMCA2 reduction, the amount of immunocomplexes was significantly smaller. It suggests that the highest level of unbound CaN was in _2 line, moderate in _3 line and the lowest in control cells. In both PMCA-reduced lines we observed similar level of PMCA4/CaN complex formation, which was however considerably lower than that of control cells. Because no such phenomenon was observed in undifferentiated cells [21], it is plausible that altered interaction of CaN with CaN-binding PMCA isoforms reported here is mandatory for the differences between cells lines. It also clearly indicates that down-regulation of PMCAs increased a pool of potentially active CaN and both “fast” PMCA isoforms could actively regulate the local CaN function. PMCA2 is a fast-reacting isoform with a high basal activity, but apart from its high affinity to Ca^2+^/CaM, is weakly stimulated by this complex [1]. PMCA4 possesses low basal activity, but Ca^2+^/CaM increases Ca^2+^ extrusion across plasma membrane several times [1, 2]. Taking into account that Ca^2+^/CaM is an activator of CaN and PMCA, not only the accessibility of this complex, but amount and affinity of both enzymes seem to be critical for determination of cell fate.

In neuronal cells differentiation process is accompanied by up-regulation of GAP43, which appears to assist neuronal pathfinding and branching during development. Interestingly, NFAT-3 was shown to be a direct transcriptional repressor of GAP43 expression [34]. At a molecular level, GAP43 is the abundant CaM reservoir in nervous cells and may concentrate calmodulin at specific sites within neurons. At steady-state, GAP43 is present in the plasma membrane of growth cone at concentrations estimated at 50–100 µM [40]. Under resting conditions (low calcium), GAP43 is bound to calmodulin, but increased Ca^2+^ concentration and phosphorylation by PKC release CaM from this complex [13]. Thus, the concerted action of PKC and CaN determines the GAP43/pGAP43 ratio, but the dynamic of this regulation seems to be cell-specific. It should be noted that both processes—phosphorylation and dephosphorylation of GAP43—are Ca^2+^-sensitive and can be regulated by even subtle changes in Ca^2+^ concentration, predominantly determined by the local PMCA composition.

We have previously reported increased expression of GAP43 and CaN in non-differentiated PC12 cells with down-regulated PMCAs isoform [21, 24]. In the present study, we also detected higher amount of these proteins in differentiated PC12 cells. It clearly indicates that up-regulation of both genes was triggered by moderate, but prolonged, elevation of Ca^2+^ and was unrelated directly to differentiation conditions. However, strong co-localization of GAP43 and CaN in differentiated PC12 cells, higher phosphatase activity in PMCA-reduced lines and a subsequent decrease in GAP43 phosphorylation index (confirmed by the assays with CsA) underlines the regulatory role of calcineurin in PMCA-conditioned differentiation process.

The next interesting result was higher level of GAP43/CaM co-localization in both PMCA-reduced lines. In addition, a diminished amount of pGAP43 may indicate lowered presence of free CaM. One can assume that less Ca^2+^/CaM complex might be formed, even if Ca^2+^ concentration elevated in these cell lines. Thus, a crucial determinant of subsequent regulation of Ca^2+^/CaM-dependent processes would be a difference in the affinity of potential effectors for this complex. In the cells, up to 300 different known targets may simultaneously compete for the same calmodulin pool altering cellular functions [9]. It was shown that they do not bind calmodulin exclusively in either Ca^2+^-saturated or Ca^2+^-free forms, but—because of unique CaM saturation kinetics—rather bind to both forms with different affinities [32, 41].

In our cell models another possible target for CaM-mediated regulation could be VGCCs. So far, a number of ion channels have been found to be modulated by CaM, with biologically important effects exerted on P/Q- and L-type channels [42–44]. Two opposite controlling mechanisms have been proposed: calcium-dependent facilitation (CDF), in which calcium influx is amplified by internal Ca^2+^ concentration and calcium-dependent inactivation (CDI) that serves as a negative feedback control system to regulate Ca^2+^ influx [45, 46]. Binding of apo-CaM to the cytoplasmic domain of the channel can effectively sense an increase in the local Ca^2+^ [47]. Ca^2+^/CaM can bind to the C-terminal region and block the calcium channel, thereby preventing the rise in cellular calcium concentration over physiological limits [48]. The CDI and CDF effects, however, do not only depend on the sequences and CaM-binding patterns, but are also finely tuned by other interaction partners in the vicinity.

The major channels expressed in differentiated PC12 cells are L- and N-types, but the presence of P/Q and T-types has been also reported [49, 50]. Our previous study has shown some differences in the expression and activity of P/Q, L and T-types of VGCCs in PMCA-reduced lines [24]. From functional assays, we have concluded that, in comparison to control cells, enlarged Ca^2+^ influx in _2 and _3 lines depended on L-type channels activity, but in _3 line significantly increased participation of P/Q-type too. Based on the results from the present study, one can assume that the negative feedback control of P/Q and L-types channels activities by cooperative action of CaM, GAP43 and CaN may represent an adaptive mechanism against calcium overload in PMCA-reduced cells. This protection seems to be more efficient in _3 line due to prominent expression of PMCA4 isoform and P/Q—type channels, as well as a result of specific regulation of their activities. It could also explain the enhanced apoptosis, which we observed in _2 line [24]. Correlation between PMCA2 down-regulation and disturbances of cell function resulting in augmented cell death has been reported in neurons, confirming protective PMCA2 role [51, 52].

It is well-documented that PMCA activity decline with age and elevated calcium leads to cell death [27, 53]. The neuronal cells are more susceptible to Ca^2+^-mediated cytotoxicity as it was shown in several neurodegenerative diseases [54]. Although the precise mechanism remains to be more versatile, our findings describe for the first time a relationship between calmodulin, GAP43, calcineurin, and probably VGCCs supportive action that would permit cells to stay alive under disturbed calcium homeostasis. In addition, these adaptive processes allowed PMCA-reduced cells to maintain their ability to growth and differentiate. Our results shed new light on the unique role of each of “fast” PMCA isoforms to control neuronal Ca^2+^ homeostasis. This approach may represent the physiological events existing in neuronal cells during aging, as well as a potential way for decreasing of neuronal cells vulnerability to neurodegenerative insults.

## Abbreviations

CaM: Calmodulin
CaN: Calcineurin
CsA: Cyclosporin A
GAP43: Growth-associated protein
NFAT: Nuclear factor of activated T-cells
PMCA: Plasma membrane calcium pump
_2: PMCA2-down-regulated PC12 cells
_3: PMCA3-down-regulated PC12 cells
VGCCs: Voltage-gated calcium channels
